# Diagnostic challenge in a 2‐year‐old boy poisoned with carbon monoxide: A case report

**DOI:** 10.1002/ccr3.8470

**Published:** 2024-02-07

**Authors:** Anahita Alizadeh, Nafiseh Pourbadakhshan, Nasrin Moazzen

**Affiliations:** ^1^ Faculty of Medicine MTRC Mashhad University of Medical Sciences Mashhad Iran; ^2^ Clinical Research Development Unit of Akbar Hospital, Faculty of Medicine Mashhad University of Medical Sciences Mashhad Iran

**Keywords:** carbon monoxide, children, diagnosis, poisoning

## Abstract

**Key Clinical Message:**

Carbon monoxide poisoning diagnosis is sometimes very difficult and should be considered in the differential diagnosis list of children's consciousness disorders even in summer.

**Abstract:**

Carbon monoxide poisoning is very dangerous, and sometimes, it is difficult to diagnose. Especially, this poisoning may have non‐specific manifestations in children and can be confused with other diseases. Here we present a 2‐year‐old child who suffered a disturbance of consciousness during the summer season. He and his family were travelers who had come to Mashhad from Kerman and were staying in a hotel room, after various investigations. It has been found that he was poisoned with carbon monoxide gas due to the leakage of carbon monoxide gas from the chimney pipe of the engine room related to the hotel's water heating into the room. After receiving oxygen and supportive treatments, he recovered and was discharged with good health. Poisoning with carbon monoxide gas is very dangerous, and the awareness of health and medical personnel in this field is essential.

## 1 INTRODUCTION

Carbon monoxide (CO) is a colorless, odorless, and tasteless gas that can cause acute poisoning in humans.^1^ CO poisoning can lead to neurological and cardiac dysfunction and can even be fatal. Children are especially vulnerable to CO poisoning due to their smaller size and immature respiratory and cardiovascular systems.^2^ In this case report, we present a case of CO poisoning in a 2‐year‐old child.

## 2 CASE PRESENTATION

One summer day, a 2‐year‐old boy weighing 12 kg was brought to the hospital due to a sudden loss of consciousness. The child along with her parents had traveled from Kerman to Mashhad the day before and were staying in one of the rooms of a hotel in Mashhad. The parents reported that the child was unconscious in their hotel room and was sent to the hospital by ambulance. The child regained consciousness on the way to the hospital. Initial examination and control of ABC showed normal findings, including SPO2: 97% and glucometric blood sugar of 87 mg/dL. The child did not have a fever or signs of infection. The history of trauma and seizures was negative. The child was growing well, and there was no history of a similar condition or previous hospitalization. There was no positive drug history, and since it was the summer season, the possibility of carbon monoxide poisoning was less important.

In the clinical and Para clinical examination, almost all the findings were normal, and the child was fully conscious in the hospital. At the insistence of the parents, they left the hospital after 4 h with self‐consent. During the 4 h of being in the hospital, the child did not have any abnormal symptoms.

About 6 h later, the child was again brought to the hospital with loss of consciousness. This time, the child had fainted as soon as he entered the hotel room and was given naloxone by *EMS personnel with suspicion of opioid consumption. The response was brief, and the child with a lethargic condition was brought to the hospital for the second time.

This time, the level of consciousness was low, and the pupils were midsized. Other tests and examinations and vital signs were normal, and SPO2 was 96%. The initial diagnosis was possible opioid poisoning. However, it was later found that both parents experienced headaches as soon as they entered the hotel room. Environmental officers investigated the hotel and found that the exit pipe of the engine room that warmed up the hotel's water was broken, and toxic gas, including CO, was pouring out of it. The hotel room was placed next to the engine room, so toxic gas was entering the room. The final diagnosis of the child's unconsciousness was CO poisoning.

## 3 TREATMENT AND MANAGEMENT

According to the history and clinical findings of the boy and his parents, doubt of CO poisoning was strong. Unfortunately, it was not possible to measure the level of carboxyhemoglobin in our center The results of the pulse oximetry device were not accurate in this case, and other relevant diagnostic equipment was not available in our center, but according to the clinical evaluation and the presence of symptoms in the parents, the possibility of the child being poisoned with carbon monoxide was very strong. Because it is not possible to use hyperbaric oxygen in our hospital and according to our country's guidelines prepared for us, carbon monoxide poisoning cases that are not very severe should be treated with oxygen and by non‐rebreathing mask for at least 4 to 6 h. So the child was treated with high‐flow oxygen therapy, which helps to eliminate CO from his body for 6 h. The child's condition improved with oxygen therapy. The child was closely monitored for cardiac and neurological dysfunction. To perform brain CT and other tests rule out other differential diagnoses and ensure that there is no recurrence of the attack or loss of consciousness, and due to the lack of laboratory facilities and the need for a comprehensive clinical investigation, we monitored the child for another day. Brain CT was normal, and ECG and troponin and creatine kinase were within normal limits. Neurologic and cardiac evaluation was normal. He was discharged after 2.5 days of hospitalization and other supportive treatments.

Because the child and his family lived in another city and returned to their city immediately after discharge, we could not perform neurological examinations and check neurological complications, but in the city where the child lives, neurological examinations for follow‐up up to 3 months after this incident were done by other physicians, and based on our telephone follow‐up, no pathological point was reported in the child from a neurological point of view.

## 4 DISCUSSION

CO poisoning is a serious condition that can lead to long‐term neurological and cognitive deficits. Children are at a higher risk of CO poisoning due to their small size and immature respiratory and cardiovascular systems. Children may be more vulnerable to carbon monoxide poisoning because of their increased metabolic rate immature CNSs and their inability to say something or understand a dangerous exposure, and newborn infants are more at risk of carbon monoxide poisoning because of the persistence of fetal hemoglobin.^1^, ^2^ CO poisoning can often be misdiagnosed as other conditions, such as opioid poisoning or viral illness.^3^ Therefore, it is important to consider CO poisoning as a possible diagnosis in cases of unexplained loss of consciousness, especially in children. Also in this group, carbon monoxide poisoning may have non‐specific symptoms and cannot be distinguished from cases such as viral infections or digestive disorders and sometimes other poisonings.^4^, ^5^ Instead, its specific diagnosis test is not always available, normal pulse oximetry results are not accurate in carbon monoxide poisoning and in most cases, possible diagnosis is based on history. Given that, measuring the level of carboxyhemoglobin in a delayed manner is usually not a useful result for a definitive diagnosis, in most cases, the diagnosis is according to history and clinical documents.^1^, ^6^, ^7^

In our settings, the diagnosis of CO poisoning is according to a history of exposure and clinical symptoms. Checking blood gases in carbon monoxide poisoning only has diagnostic value when a co‐oximeter is available, but this device is not available in our center, also it is not possible to measure carboxyhemoglobin and other tests, including measuring biomarkers related to oxidative stress.^8^ Therefore, based on clinical suspicion and existing symptoms, and sometimes the presence of similar symptoms in the patient's companions and history of exposure, we suspect or diagnose carbon monoxide poisoning. In the present case, the child's parents had also similar symptoms. Evidence of carbon monoxide gas leakage into the patient's residence was also found. The clinical symptoms were consistent with the diagnosis.

Therefore, the child was diagnosed with carbon monoxide poisoning and underwent the aforementioned treatments and the condition improved.

Also after carbon monoxide poisoning, there is a possibility of neurological symptoms, including persistent neurological symptoms that last for more than 3 months, as well as delayed neurological symptoms that reoccur after a period of recovery. According to some available sources, the rate of late neurological complications is reported to be around 40% in adults and 3%–17% in children. In some articles, persistent neurological complications in children have been reported in about 10%.^2^

Therefore, after the child's discharge, we followed up on the child's neurological complications over the phone. Fortunately, he did not suffer from persistent or late neurological complications.

## 5 CONCLUSION

Carbon monoxide poisoning is very dangerous, and sometimes, it is difficult to diagnose, and it is necessary for physicians to consider this diagnosis in the differential diagnosis of any sudden consciousness disorder in children and to be familiar with the necessary measures in this case.

## AUTHOR CONTRIBUTIONS

**Anahita Alizadeh:** Conceptualization; resources; writing – original draft. **Nafiseh Pourbadakhshan:** Conceptualization; writing – original draft. **Nasrin Moazzen:** Validation; writing – review and editing.

## FUNDING INFORMATION

There is no funding for this article if required, and the information about the article is readily available for use.

## CONFLICT OF INTEREST STATEMENT

None.

## ETHICS STATEMENT

Ethics standards have been observed. Consent has been obtained from the legal guardian of the child to write this article.

## CONSENT

Written informed consent was obtained from the patient to publish this report in accordance with the journal's patient consent policy.

## Data Availability

The information related to the article is available, and if necessary, you can use the information.
